# Perceived organizational culture and its relationship with job satisfaction in primary hospitals of Jimma zone and Jimma town administration, correlational study

**DOI:** 10.1186/s12913-020-05319-x

**Published:** 2020-05-19

**Authors:** Dereje Mesfin, Mirkuzie Woldie, Ayinengida Adamu, Fitsum Bekele

**Affiliations:** 1 Department of Public Health, College of Medicine and Health Sciences, Wolkite University, Wolkite, Ethiopia; 2 Department of Health Economics, Policy and Management Institute of Health Science, Jimma University, Jimma, Ethiopia

**Keywords:** Organizational culture, Job satisfaction, Jimma zone, Hospital management

## Abstract

**Background:**

The concept of Organizational Culture (OC) which refers to the pattern of values, norms, beliefs, attitudes and assumptions may not be articulated through verbal language. However, it shapes the way people behave and the way things get done in an organization. The management of organizational culture is increasingly viewed as necessary part of health system reform. Major cultural transformation of an organization must be secured alongside structural and procedural changes in order to achieve desired quality and performances improvements in health systems. It is therefore essential to understand organizational culture, job satisfaction level of the health workers and the link between them.

**Methodology:**

Facility based cross sectional study was conducted in four primary hospitals of Jimma zone and town administration. A self-administered questionnaire was used to collect the data. The collected data were checked for completeness, entered and documented into Epi-data version 3.1 and Exported to SPSS version 21 for analysis*.* Finally descriptive statistics, Paired t-test and multiple linear regression analysis were used to assess the relationship between organizational culture and job satisfaction and the results were presented using tables and charts.

**Result:**

It was indicated from the finding that, the dominant existing organizational culture typology in the primary hospitals was Hierarchy culture (MS = 22.31, ±2.82).and the preferred organizational culture typology was Innovative culture (MS = 26.09, ±4.72). The health workers had low to medium level of job satisfaction where only (29.40%) of the health workers were very satisfied with their hospital physical working environment. Existing perceived clan culture had positive and significant correlation with health workers’ satisfaction in relation to work relation dimension (*r* = .16, *p* < 0.002).

**Conclusion:**

while acknowledging all limitation of observational study we reached to the conclusion that an employees of the respective primary hospitals would prefer to work in environment characterized by innovative and clan culture and their satisfaction level is medium so that the managers should undertake major cultural transformation and must work to improve the job satisfaction level of health workers within their respective hospitals.

## Background

Organizational culture (OC) is a difficult concept to define concretely. Different authors define it differently [1]. Armstrong and Michael defined it as; Organizational culture is the pattern of values, norms, beliefs, attitudes and assumptions that may not have been articulated in words but shape the ways in which people behave and things get done in the organization [2].

According to Schien Edger, organizational culture is the pattern of shared basic assumptions invented, discovered or developed by a given group as it learns to cope with its problems of external adaptation and internal integration that has worked well enough to be considered as valid and therefore to be taught to new members as the correct way to perceive, think and feel in relationship to those problems [3].

Organizational culture refers to the beliefs and values that have existed in an organization for a long time, and to the beliefs of the staff and the foreseen value of their work that will influence their attitudes and behavior [4].

Organizational culture has been receiving extra attention ever since the concept was proposed by American scholars four decades back. The Academicians and managers have reached the consensus that organizational culture is the core competency for an organization and on the fact that it will impact effectiveness of the individual workers, groups and the organization as a whole [5].

As sited by Bandana et al. in 1988 organizational culture is a multidimensional construct, and therefore it is essential to evaluate each of its dimensions during the study of the culture of a given organization. With this regard when we discuss the culture of a given organization we are referring to multiple typologies or dimensions of organizations culture rather than one composite culture derivative [6].

Apart from the various types and models that have been suggested for the description of organizational culture, quite a big number of instruments for the measurement of organizational culture have been developed as well. In the last few decades, both academics and practitioners in management science have focused on studying possible ways to measure the dimensions of organizational culture and how such measures relate to the effectiveness and competitiveness of an organization [7]. Methods developed by Quinn and Rohrbaugh’s [8], Hofstede Organizational Culture Questionnaire [9], the one developed by Denison [10] etc. are among the most complete and elaborated ones.

The Competing Values Framework (CVF) has been widely used in health services research to assess organizational culture as a predictor of quality improvement implementation, employee and patient satisfaction and team functioning among other outcomes [11].

The four effectiveness criteria models in the CVF are called four organizational culture typologies. Cameron and Quinn Based on former organizational culture studies in the literature termed these four culture typologies as Clan, Adhocracy, Market, and Hierarchy. The implications of each culture type are summarized as follows [12].

### The clan culture

The clan culture is an atmosphere of collectivity and mutual help with an emphasis on empowerment and employee evolvement. Typical characteristics of clan-type firms were teamwork, employee involvement programs and corporate commitment to employees.

### The adhocracy culture

A major goal of the adhocracy culture is to foster adaptability, flexibility, and creativity where uncertainty, ambiguity, and information overload are typical. An important challenge for these organizations is to produce innovative products and services and to adapt quickly to new opportunities.

### The market culture

The term market is not similar to the marketing function or with consumers in the market place. Rather, it refers to a type of organization that functions as a market itself. It is oriented towards the external environment instead of internal affairs.

### The hierarchy culture

The hierarchy culture has a clear organizational structure, standardized rules and procedures, strict control, and well defined responsibilities.

In the Health sectors, there are different kinds of employees such as health employees, administrative employees and technical personnel and other categories that perceive culture of the health organization in different ways and have their performance and job satisfaction affected in different ways [13].

The management of organizational culture is increasingly viewed as necessary part of health system reform. Major cultural transformation of an organization must be secured alongside structural and procedural changes in order to achieve desired improvements in quality and performance of the health system [14].

Due to its impact on the different aspects of an organization, Organizational culture is a specific research topic in management knowledge which has never lost popularity in its research field. Besides, it is considered as a key factor for studying different aspects of organizational life and research questions regarding its impact on organizational goals are increasing from time to time [15].

Defining Organizational climate is not an easy task because it is about the perception of the employees which is difficult to measure in concrete sense and varies from one employee to the other; it can be considered as the combination of psychological phenomena, which are the perceptions of individuals about their work place it is represents the condition of the organization’s culture. Organizational climate has a strong influence on employee attitudes regarding their sense of personal relationship, belongingness and commitment to work [16].

Historically organizational culture and climate were sometimes seen as distinct organizational variables; sometimes as arising from differing academic traditions; and sometimes as rival theoretical concepts. Alternatively, they have been viewed as two sides of the same coin, reflecting similar manifestations of the way things are done [17].

Until the past two decades or so, there have also been signficant differences in the methods used to study climate and culture. So, climates have been characterized by employee surveys and culture by qualitative case studies. A historical review of the climate and culture literature, however, reveals that, recently culture has been much more studied using surveys. Also, the issues addressed by culture studies can overlap and at the same time be considerably different from the issues addressed by climate surveys [18].

Because of its ability to create sense of identity and rules that help the organization to achieve its goal, culture can be a powerful determinant of long-term firm success. This means, a good hospital culture leads to better individual performance than conventional training. Besides, the knowledge of the culture of an organization is an effective weapon to cope a fast changing environment, since knowledge is likely to offer solutions to the problems of the health organization [19, 20].

Job satisfaction is the extent one feels positively about his or her job [21]. It is employee’s evaluation of job responsibilities and the working environment. Based on this evaluation, an employee may develop some positive or negative attitude towards the organization’s shared rules, beliefs and values which strongly influence their working conditions. Hence, this positive or negative response to job evaluation is worth discussing because its importance for organizational well-being and success [22].

Employee satisfaction can be predicted from specific organizational culture. The nature of culture installed in an organization potentially derives employee feeling at work place which could result in noticeable variation of satisfaction levels. This notion leads to the definition of employee satisfaction in association with organizational culture. It also implies that when cultural values of an organization match employee’s expectations, employees feel satisfied and vice versa [23].

Although literature said organizational culture in general has been studied extensively, there are only few studies in health care settings. Moreover, the quantitative studies that have been conducted on organizational culture have generally been conducted in the developed countries and little is known about the organizational culture of countries with different socio-economic conditions [24].

Ethiopia is one of developing countries which have different socio-cultural and economic context from the above mentioned countries and the organizational culture of health institutions in the study area is not studied before so this study will try to fill the information gap on the organizational culture of health institutions and will try to explore its effect on the job satisfaction it employee in primary hospitals of Jimma zone south Western Ethiopia.

The objective this study is to understand healthcare workers perception of organization culture and its correlation with their job satisfaction level and also it will try to pinpoint specific dimensions of strong culture within the primary hospitals and area of weakness which will require culture change strategies in order to improve the performance of the employees.

The results can be used as a ground for the successful implementation of quality improvement programs, including clinical governance and performance at hospitals. Moreover, executives with knowledge of the organizational culture of their hospital can try to remove possible errors and prepare hospitals to implement quality improvement programs and changes successfully.

The findings of the study will serve as reference for academician and researchers who have interest to conduct researches on organizational culture.

## Methods

### Study design

Facility based cross-sectional quantitative study was conducted in primary hospitals of Jimma zone.

### Study area and period

The study was conducted in all four primary hospitals found in Jimma zone and town administration named as Shenen Gibe, Seka, Agaro and Limu Genet. Shenen Gibe Hospital is the youngest hospital which was established in 2012 and currently provides services for both in patients and out patients. It has 50 inpatient beds with different departments including Medical, Surgical, Pediatrics wards, Obstetrics and Gynecology [25]. Limu Genet Hospital is a primary hospital found in Limu-Genet town that is located 80 km south of Jimma town [26]. There were total of 731 health workers who are eligible for this study working in the four hospitals.

### Population

#### Source population

All health workers who were working in primary hospitals of Jimma zone and Jimma town administration, south west of Ethiopia were the source populations of this study.

#### Study population

The study population was determined by using single population proportion formula which resulted with final sample size of 383 and the study participants were selected using simple random sampling technique after proportional allocation to each hospital.

### Eligibility criteria

#### Inclusion criteria

Health workers who were permanently employed in the respective hospitals for at least one year and above were included in the study.

### Study variables

#### Dependent variables

Health workers job satisfaction level as measured by the following satisfaction dimension scores.

- Health workers job satisfaction in relation to hospital physical working environment
- Health workers job satisfaction in relation to supervision
- Health workers job satisfaction in relation to communication
- Health workers job satisfaction in relation to work relation

#### Independent variables

- Sociodemographic Characteristics

Age, gender, educational status, job category, managerial responsibility, net monthly salary and area of residence, year of experience …

- Existing Organizational culture typology as measured by the following dimension scores
  - Perceived existing clan culture score
  - Perceived existing innovative culture score
  - Perceived existing market culture score
  - Perceived existing hierarchy culture score

### Data collection instrument and procedures

#### Instruments

The study instrument had three parts.

Part one assessed Socio-demographic characteristics of the respondents. Part two of the instrument measured health workers perception of organizational culture using organizational culture assessment instrument (OCAI) adapted from Cameroon and Quinn which is measures organizational culture using four dimensions named as Clan, Adhocracy, Market, and Hierarchy (see the introduction part). at both existing and preferred dimensions [12]. The instrument is consisted of twenty-four declarative items arranged in six domains which are Dominant Characteristics, Organizational Leadership, Management of Employees, Organization Glue, Strategic Emphasis and Criteria of Success. The third part of the questionnaire was about job satisfaction which was adapted from Joe Kavanaugh et al. [27]. The tool had 36 five-point Likert scale items within five domains which are Feelings about the hospital, support for quality. Supervision, communications and working relations.

#### Data collection techniques

Self-administered structured questionnaires were used for data collection. Data collection facilitators distributed the questionnaires to all eligible health workers at the same time. After informing participants to fill the questionnaires privately, it was disseminated during lunch breaks and/or at entry times like early morning. Again, written guidelines were prepared and provided to the data collectors to assure that each health workers received the same direction and information.

#### Data collectors and supervisors

Twelve data collectors who had diploma in health related fields with or without previous experience in data collection were selected for data collection. However, data collectors were fluent particularly in local and English languages which added for the successful completion of the process.

Four supervisors with Bachelor of Science degree in health related fields and with previous experience as a supervisors of data collection were recruited from the nearby health centers to supervise the data collection process.

### Data quality control

#### Before data collection

A one-day orientation was given for both the above mentioned data collectors and the supervisors to explain the objectives of the study, the contents of the questionnaire, issues related to the confidentiality of the responses, the rights of respondents and the data collection processes.

To ensure the clarity and validity of the instruments, the questionnaires were pretested on 5 % of the sample size in Jimma university specialized hospital before the study was undertaken by the actual data collection site. It was observed from the pretest that all of the questionnaires were answered and returned to the data collectors but the participants taken more longer time than expected while filling the questionnaire, to that end the respondents were directly contacted and asked about the clarity of the questions in the instrument and some modifications were made on wording and arrangement of the questions in a way that make them more understandable for the study participants. Besides, the data collectors were also asked if there was any kind of difficulty on the data collection process like the timing of data collection the patient load and the like. Accordingly, changes were made on the data collection time in a way which enables them to contact the healthcare workers in more comfortable way i.e. to the periods with low patient flaws.

#### During and after the data collection

Respondents were reassured of the confidentiality of the data and informed of the fact that there is no preferred or correct answer. All the questionnaires filled by the respondents were checked regularly by four of supervisors assigned to each primary hospitals and the principal investigator for completeness and fortunately all the returned questionnaires were complete.

### Procedure of data analysis

The collected data were checked for completeness, edited, cleaned, entered and documented in EpiData program version 3.1., then exported to SPSS version 21.0 for analysis. After data were checked for inconsistencies and missing values, descriptive statistics such as; mean, frequency and percentage were calculated for Sociodemographic variables and presented using tables and charts.

Basic assumptions underlying factor analysis such as; factorability of the data, Pearson’s correlation coefficient of greater than 0.30 (at least for two items) or greater, the level of significance of the Bartlett’s test of sphericity, the value of the Kaiser-Meyer-Olkin measure of sampling adequacy all > 0.50 were checked. Components with Eigen-value greater than one, factor loading greater than 0.4 and Cronbach’s alpha > 0.7 were considered for further analysis and all assumptions of linear regression were also checked.

Principal components analysis with varimax rotation was used to extract the measurement scales for the dimensions of organizational culture and job satisfaction. Cronbach’s alpha was used to assess the internal reliability of the extracted components.

Uni-variate analysis such as; mean score and percentage maximum scale scores were calculated for the dimensions of organizational culture and job satisfactions and presented in tables.

Dependent (paired) Samples T-test was used to see if there was statistically significant difference between existing and preferred organizational culture typologies in the primary hospitals at (*p* < 0.05) and the result was presented in tables. Pearson correlation analysis was conducted to see the linear relationship between organizational culture and job satisfaction.

### Operational definitions and measurement

To describe organizational culture typologies as more dominant and/or less dominant for both the existing and preferred organizational culture dimensions, single index score in the form of overall mean scores was calculated. Accordingly, it was (19.42 ± 2.44) for existing organizational culture and (23.19 ± 3.62) for preferred organizational culture. Organizational culture typologies with mean scores above this index were considered as highly dominant culture and those with mean sores below the index value were considered as low dominant culture [28].

Job Satisfaction levels were calculated based on the percentage of maximum scale scores. The percentage of maximum scale scores was computed using the following formula.

$$ \mathbf{Percentage}\ \mathbf{maximum}\ \mathbf{scale}\ \mathbf{score}=\left(\mathrm{Actual}\ \mathrm{score}-\mathrm{potential}\ \mathrm{minimum}\ \mathrm{score}\right)/\left(\mathrm{potential}\ \mathrm{maximum}-\mathrm{potential}\ \mathrm{Minimum}\right)\ \mathrm{x}100 $$

Feyissa et al. [29] and Deriba et al. [30] accordingly job satisfaction is termed (as)

Low = if the percentage maximum scale score of the dimension is ≤5%

Medium = if the percentage maximum scale score of the dimension is between 51 and 70%

High = if the percentage maximum scale score of the given dimension is ≥71%

## Results

### Sociodemographic characteristics

The result presented in this section is based on the data collected from 326 participants which indicate 86% of total response rate. Among the respondents, 154(47.20%) were males and the rest were females. Also, most of the respondents belong to the age category of 25–29 (50.90%) and few of them were above 36 (7.70%). Out of the total of 326 health workers, 22 (6.70%) of them were physicians, 128(39.30%) were nurses, 88 (27.00%) other technical staffs and 88 (27.00%) of them were supportive staffs. Regarding positions (managerial duties), out of the total participants, 39(12.00%) have managerial responsibility and the rest 287 (88.00%) have no any managerial responsibility. Among the study participants, 313 (96.10%) had work experience of between 2 and 5 years (see Table 1).

**Table 1:** Sociodemographic characteristics of health workers in primary hospitals of Jimma zone and town administration, southwest of Ethiopia April, 2017(*n* = 326)

Sociodemographic Characteristics		Frequency	Percent
Sex	Female	172	52.76
	Male	154	47.23
Managerial responsibility	No	287	88.00
	Yes	39	12. 00
Educational status	Bachelor degree	155	47.54
	Diploma (10 + 3)	140	42.94
	< 12th grade	27	8.28
	≥Master’s	4	1.22
Job category	Nurse	128	39.6
	Physician	88	26.99
	Other technical staff	88	26.99
	Supportive staff	22	6.74
Year of service	2-5 years	313	96.01
	> 5 years	13	3.98

### Perceived organizational culture

#### Perceived existing organizational culture

In the primary hospitals of Jimma zone and Jimma town administration, Hierarchy culture with mean score of (22.31, ±2.81) was the dominant existing organizational culture typology. Then, market culture was the secondly dominant culture type; (M = 19.21, ±3.38) (Table 2).

**Table 2:** Existing organizational culture typologies in primary hospitals of Jimma zone and town administration, southwest of Ethiopia April, 2017(*n* = 326)

Scales	N	Minimum	Maximum	Mean raw score	PMS
Clan	326	10	29	18.98(3.55)	51.10%
Innovative	326	9	25	17.17(3.21)	50.49%
Market	326	10	28	19.21(3.38)	58.48%
Hierarchy	326	14	29	22.31(2.81)	69.28%

*MS* Mean score, *PMS* percentage maximum scale score

#### Perceived preferred organizational culture

According to the findings of this study, innovative culture; (MS = 26.09, ±4.72) was the dominantly preferred organizational culture typology and hierarchy culture (MS = 21.59, ±3.75) was the least preferred culture type in the Primary hospitals of Jimma zone and Jimma town administration (Table 3).

**Table 3:** Descriptive statistics of perceived preferred organizational culture typologies in the Primary Hospitals of Jimma zone and Jimma town administration, southwest of Ethiopia, April 2017

Culture types	N	Min.	Max.	MS (SD)
Clan culture	326	13	30	22.4(3.70)
Innovative culture	326	10	35	26.0(4.70)
Market culture	326	10	30	22.5(3.50)
Hierarchy culture	326	12	30	21.5(3.70)

### Difference between existing and preferred organizational culture typologies

To see if there were significant differences between existing and preferred organizational culture typologies in the primary hospitals paired t-test was employed. Accordingly, there was a significant difference between all pairs of existing and preferred culture dimensions (Table 4).

**Table 4:** Comparison between the dimensions of existing and preferred organizational culture in the Primary hospitals of Jimma zone and town administration, Southwest of Ethiopia, April, 2017

Culture types	Existing		Preferred		MD	t	*P*-value
	MS	SD	MS	SD			
Clan	18.98	3.55	22.4008	3.73	−3.50	−12.15	.000
Innovative	17.17	3.21	26.09	4.72	−8.92	−29.26	.000
Market	19.21	3.38	22.58	3.58	−3.37	−13.50	.000
Hierarchy	22.31	2.81	21.59	3.75	.72	2.971	.003

### Perceived job satisfaction level of health workers

Perceived satisfaction with work relation (PMS = 55.48%) was the dimension with the medium level of health workers job satisfaction. Followed by perceived satisfaction with communication dimensions (PMS = 50.38%) (See Table 5).

**Table 5:** Perceived job satisfaction level of Health workers in the Primary Hospital of Jimma zone and Jimma town administration, southwest of Ethiopia. April, 2017

Extracted components	N	Min	Max	MRS	PMS
Hospital physical working environment	326	5.00	25	14.4(4.4)	47.13%
Satisfaction with supervision	326	3.00	15	9.01(2.7)	50.10%
Communication	326	3.00	15	9.04(2.7)	50.38%
Work relation	326	2.00	10	6.43(2.0)	55.48%

*MS* Mean score, *MRS* mean raw score, *PMS* percentage maximum scale score

### Relationship between organizational culture and job satisfaction

According to the findings of our study all of the organizational culture dimensions had positive and significant correlation with hospital physical working environment (see Table 6).

**Table 6:** Correlation between perceived existing organizational culture and Health workers job satisfaction in the Primary Hospital of Jimma zone and town administration, southwest of Ethiopia. April, 2017

		hospital working environment	Supervision	Communication	work relation
Clan	r	.21^**^	.13^*^	.018	.16^*^
	Sig.	.000	.017	.752	.002
Innovative	r	.15^*^	.08	−.06	.19^**^
	Sig.	.004	.128	.259	.000
Market	r	.32^**^ .000	.19^*^	.14*	.25^**^
	Sig.		.001	.008	.000
Hierarchy	R	.20^**^	.17^*^	.15^*^	.17*
	Sig.	.000	.002	.005	.002

(**Pearson Correlation is significant at the *P* < 0.001 level,*Correlation is significant at the *P* < 0.05 level)

Health workers who perceived clan culture as the existing culture typology in their hospital are found to be better satisfied with hospital working environment compared to those who perceived their hospital culture is more of innovative. Those health workers who perceived their hospital culture is innovative are strongly satisfied with the work relation in the hospital than those who believe on the prevalence of clan culture but less satisfied with the work relation compared to those health workers who perceived their hospital culture is predominantly of market type. Again those health workers who perceived the existence of market culture in their hospitals found to be better satisfied with the work relation in their respective hospitals compared to those who perceived hierarchy culture as the dominant culture typology in the hospitals, in addition health workers who believe that their hospital follows, Hierarchy culture found to be more satisfied with the communication structure and the supervision compared to those who perceive that the their hospital culture is more of a clan type.

## Discussion

### Perceived organizational culture

According to the findings of this study, hierarchy culture, which is characterized by formal rules, procedures and controlling management style was the dominant existing culture typology in the primary hospitals of Jimma zone and Jimma town administration with the mean score of (MS = 22.31, ±2.82). Seemingly, market culture was the second dominant culture type with mean score; (MS = 22.58, ±3.38). These findings are similar with the study conducted in Greek where, the dominant existing organizational culture type was hierarchy culture (MS = 39.2, ±20.48) followed by market culture (M = 23.49, ± 7.76) (48). Another study conducted in Libya also showed that the dominant existing culture type was hierarchy (MS = 3.03, ±0.73) followed by clan culture type [31]. Hence, the primary hospitals of Jimma zone and Jimma town administration can be described as work places of predominately characterized by hierarchy and market culture.

In the primary hospitals of Jimma zone and town administration, Innovative culture which is characterized by creativity, professional freedom and transformational leadership is the most preferred culture type by the health workers (MS = 26.09, ±4.72). Likewise, in the study from Greek, the preferred organizational culture was innovative culture followed by clan culture type which is characterized by team work, capacity development and nurturing leadership [32]. But in the General Hospital of Larissa [13], the most preferred culture type was hierarchy one which is characterized by prominence of rules, regulations, working and reporting criteria. The difference between the two areas might be due to experience difference between the two hospitals since hierarchy culture is typical as organizations become more experienced [31].

Therefore, it is possible to say that the study revealed a significant mismatch between the perceived existing organizational culture types and the preferred culture types by the health workers. They preferred hierarchy culture to be less dominant and they would like to work in an environment dominated by innovative and clan culture where there is creativeness and strong interpersonal interaction respectively. So, this result was consistent with the findings particularly from Greek [32]. In both cases, the participants responded that they want clan and innovative culture to be more dominant in their organization and hierarchy, culture to be less dominant in the future.

### Level of job satisfaction

According to health workers job satisfaction assessment conducted in four primary hospitals, only 29.40% of the health workers were very satisfied with their hospital physical working environment. This level of satisfaction was very low compared to the levels reported from Guangdong province; China [33]. This gap might be due to the infrastructure difference between the two areas.

In this study, among the total health workers, 20.25% of them were very satisfied with work relation in the hospitals. When we compare this particular result with findings obtained from North Shoa, Ethiopia, it is higher, for only 10.20% health professionals were found to be very satisfied with their work relation [34]. This gap might be due to socio-cultural disparity between the two areas. But when we compare it with the study conducted in Kenya where, majority of health workers were highly satisfied with colleagues [35], it was lower. This might be due to short lifespan (run-time) of the hospitals in the two areas. In the case of our study areas, the age of the hospitals is short that, a strong relation between the health workers is yet to be developed.

Among health workers in our study area, 44.80% reported that they were satisfied with the supervision activity in their hospitals. When we compare this with the finding in china where 84.90% were satisfied with their supervisors [33], the level of satisfaction is lower. The reason behind this disparity might be due to difference in experience among the supervisors.

### Correlation between perceived existing organizational culture and job satisfaction level

Existing perceived clan culture had positive and significant correlation with health workers’ satisfaction in relation to work relation dimension (*r* = .168, *p* < 0.002). But according to previous study conducted in Zahedan University of Medical Sciences, Iran, clan culture was found to have negative correlation with the satisfaction with co-workers (*r* = − 4.1, *P* < 0.000) [36]. This difference might be due to the difference in the type of work performed in the two study areas i.e. the type of work in hospitals (like the case our study) might require more of team approach but academics (like the case in Zahedan University) may prefer rather individual competence.

## Limitation of the study

Since the study is cross-sectional type it is difficult to set strong causation among the independent and dependent variables in addition to that desirability bias might affect the quality of the data more over since the data is collected through questionnaire survey, our work is subjected to information bias which is the intrinsic nature of the data collection instrument, the other limitation is that the study should have been implemented by qualitative data collection methods.

## Conclusion

The dominant perceived existing organizational culture in the primary hospitals of Jimma zone and town administration was hierarchy culture. But the least existing organizational culture type was innovative culture.

Innovative culture was the most preferred culture type followed by market culture. But Hierarchy culture type was the least preferred culture type by the health workers.

There was a significant difference between the perceived existing organizational culture types and the preferred culture types in the primary hospitals of Jimma zone and Jimma town administration.

The job satisfaction level of health workers in the primary hospitals was medium in relation to supervision, communication and work related dimensions. However, it was low in relation to hospital physical working environment. Therefore the hospital administration must work hard to improve infrastructures of the hospital including staff recreation places and the likes; furthermore perceived existing Clan culture was positively correlated with health workers job satisfaction so that the administration of the hospital should work towards strengthening social interaction of the employees.

## Data Availability

Data will be available from the authors on justified request.

## Abbreviations

FMOH: Federal Ministry of Health
HMIS: Health Management information system
MS: Mean score
LICs: Low income countries
OC: Organizational culture
OCAI: Organizational culture assessment instrument
PATH: Performance assessment tool for quality improvement in hospitals
PCA: Principal Component analysis
PMS: Percentage mean score
RMS: Raw mean score
WHO: World Health Organization
